# Telocytes in Crohn’s disease

**DOI:** 10.1111/jcmm.12177

**Published:** 2013-11-19

**Authors:** Anna Franca Milia, Martina Ruffo, Mirko Manetti, Irene Rosa, Dalila Conte, Marilena Fazi, Luca Messerini, Lidia Ibba-Manneschi

**Affiliations:** a Department of Experimental and Clinical Medicine, Section of Anatomy and Histology, University of FlorenceFlorence, Italy; b Department of Surgery and Translational Medicine, University of FlorenceFlorence, Italy; c Department of Experimental and Clinical Medicine, Section of Surgery, Histopathology and Molecular Pathology, University of FlorenceFlorence, Italy

**Keywords:** telocytes, Crohn’s disease, interstitial cells of Cajal, ileum, fibrosis, CD34, immunohistochemistry

## Abstract

Crohn’s disease (CD) is a relapsing chronic inflammatory disorder that may involve all the gastrointestinal tract with a prevalence of terminal ileum. Intestinal lesions have a characteristic discontinuous and segmental distribution and may affect all layers of the gut wall. Telocytes (TC), a peculiar type of stromal cells, have been recently identified in a variety of tissues and organs, including gastrointestinal tract of humans and mammals. Several roles have been proposed for TC, including mechanical support, spatial relationships with different cell types, intercellular signalling and modulation of intestinal motility. The aim of our study was to investigate the presence and distribution of TC in disease-affected and -unaffected ileal specimens from CD patients compared with controls. TC were identified by CD34/PDGFRα immunohistochemistry. In affected CD specimens TC disappeared, particularly where fibrosis and architectural derangement of the intestinal wall were observed. In the thickened muscularis mucosae and submucosa, few TC entrapped in the fibrotic extracellular matrix were found. A discontinuous network of TC was present around smooth muscle bundles, ganglia and enteric strands in the altered muscularis propria. At the myenteric plexus, the loss of TC network was paralleled by the loss of interstitial cells of Cajal network. In the unaffected CD specimens, TC were preserved in their distribution. Our results suggest that in CD the loss of TC might have important pathophysiological implications contributing to the architectural derangement of the intestinal wall and gut dysmotility. Further functional studies are necessary to better clarify the role of TC loss in CD pathophysiology.

## Introduction

Crohn’s disease (CD) is a complex chronic inflammatory disorder of the gastrointestinal (GI) tract, formerly described and classified as a form of segmental ileitis 1. Although the aetiology is still unknown, it is now widely accepted that CD is caused by genetic susceptibility, abnormal intestinal permeability, interaction between microbiota and surface epithelial cells, dysbiosis and dysregulation of the host’s immune response. The incidence and prevalence of the disease in the developed world have markedly increased in the last decades 2–3.

Crohn’s disease is characterized by a segmental and transmural bowel wall relapsing inflammation that waxes and wanes over years, leading to the disruption of the normal architecture of the mucosa and loss of the regular disposition of the glands. Lymphoid aggregates, granulomas and giant multinucleated cells are peculiar of the disease. Fibrosis, obstructive lymphocytic lymphangitis, lymphoedema and critical luminal narrowing may follow the inflammation, leading to intestinal stenosis.

The behaviour of CD substantially varies during the course of the disease, whereas its anatomical location is mostly stable 4. Crohn’s disease is located in the terminal ileum in 45%, colon in 32%, ileocolon in 19%, and upper GI tract in 4% of cases 5. To date, no resolving therapy is available. Medical treatment consists mainly of symptomatic and supportive care, but it is still considered far from satisfying, and surgical treatment has still a high incidence 2–5.

In CD, evidence of bowel dysmotility has been reported at different levels of the GI tract 6. Motility of the GI tract involves complex processes that require the structural integrity and functionality of different cellular elements. Indeed, mechanical activity of smooth muscle cells is in part controlled by the enteric nervous system and by the interstitial cells of Cajal (ICC). In this regard, morphological studies have reported severe damages of the enteric neural structures and a reduced population of ICC in CD patients 6.

Different interstitial cell types have been identified in the GI tract, each forming networks within the neuromuscular compartment:

- The ICC that are positive for c-kit (CD117) and negative for CD34 and platelet-derived growth factor receptor α (PDGFRα) 7–8. These cells are considered the pacemaker cells and the principal mediators of neurotransmission in the GI tract 8–9.
- The telocytes (TC) that are positive for CD34 and PDGFRα and negative for c-kit 7,8. These cells, formerly identified as CD34-positive interstitial cells or PDGFRα-positive ‘fibroblast-like’ cells different from ICC in several publications, have also been proposed to play an important role in the enteric neurotransmission 7–12.

Telocytes have been recently described in many organs of humans and laboratory mammals (http://www.telocytes.com) 13,14, including the GI tract 8,16. Telocytes are ultrastructurally characterized by a small cell body and extremely long processes, termed telopodes, which display a moniliform aspect alternating thin segments (podomers) with dilated regions (podoms) 18. Transmission electron microscopy is the best available method for TC identification. By immunohistochemistry, TC do not express specific marker(s). Indeed, immunohistochemical results appear to be different according to the organ and/or the animal species examined, and it has also been suggested that such differences might be at the basis of region-specific TC roles 18. However, at present, CD34 labelling remains the best available choice for the immunohistochemical identification of TC 13–18. In fact, by immunoelectron microscopy it has been demonstrated that in the human gut the CD34-positive interstitial cells are TC 7. Moreover, recent studies have described the gene expression profile, microRNA signature and electrophysiological characteristics of TC from different organs 19,20. Several roles have been proposed for TC, but none of them has been fully established yet 18–22. It has been suggested that TC might have a role (*i*) as a mechanical support to drive the correct organization of the connective tissue within organs 18; (*ii*) in intercellular signalling between stromal cells, immune cells, smooth muscle cells, microvessels and nerve bundles by cell-to-cell contacts and paracrine secretion of signalling molecules 13–24; (*iii*) in the neurotransmission by spreading the slow waves generated by the pacemaker ICC 8–18.

As GI dysmotility with ICC defects is a peculiar pathological feature of CD 6–25, the aim of our study was to investigate the presence and distribution of TC in surgical specimens obtained from disease-affected and -unaffected ileal segments of CD patients.

## Materials and methods

### Surgical specimens

Full-thickness samples of terminal ileum were obtained from CD patients (*n* = 55; mean ± SD age 41.3 ± 10.2 years) who underwent surgery for intestinal obstructive stenosis. The diagnosis was based on conventional clinical and endoscopic criteria and on the histopathological analysis of biopsies and resected preparations. Specimens were taken from macroscopically disease-affected segments, characterized by the typical inflammatory lesions of the mucosa, and from macroscopically disease-unaffected mucosal segments.

Full-thickness control samples of the same anatomical region were obtained from patients who underwent surgery because of intestinal cancer (*n* = 20; mean ± SD age 61.3 ± 9.5 years). Specimens were carefully taken at least 8 cm from the margins of the tumour, in areas that appeared to have no inflammatory or neoplastic infiltration according to macroscopic observation. Histopathological analysis was performed to further exclude the presence of inflammatory and neoplastic infiltration. All samples were collected at the archives of the Section of Surgery, Histopathology and Molecular Pathology and Section of Anatomy and Histology, Department of Experimental and Clinical Medicine, University of Florence.

The specimens were fixed in 10% buffered formalin, dehydrated in a graded ethanol series and xylene, and embedded in paraffin. Sections were cut (5 μm thick) using a Leica RM2255 rotary microtome (Leica Microsystems, Mannheim, Germany), deparaffinized and stained with haematoxylin–eosin for routine histology and with Masson’s trichrome with aniline blue (catalogue no. 04-010802; Bio-Optica, Milan, Italy) to detect the presence of fibrosis. Tissue sections were observed under a Leica DM4000 B microscope (Leica Microsystems), and transmitted light images were captured by a Leica DFC310 FX 1.4-megapixel digital colour camera equipped with the Leica software application suite LAS V3.8 (Leica Microsystems).

### Immunohistochemistry

After deparaffinization, tissue sections were boiled for 10 min. in sodium citrate buffer (10 mM, pH 6.0) for antigen retrieval and treated with 3% H_2_O_2_ in methanol for 15 min. to block endogenous peroxidase activity. Sections were then washed and incubated with Ultra V block (UltraVision Large Volume Detection System Anti-Polyvalent, HRP, catalogue no. TP-125-HL; LabVision, Fremont, CA, USA) for 10 min. at room temperature according to the manufacturer’s protocol. After blocking non-specific site binding, slides were incubated overnight at 4°C with mouse monoclonal anti-human CD34 (1:50 dilution; clone QBEnd-10, catalogue no. M7165; Dako, Glostrup, Denmark) or rabbit polyclonal anti-α-smooth muscle actin (α-SMA; 1:100 dilution; catalogue no. ab5694; Abcam, Cambridge, UK) antibodies, diluted in 1% bovine serum albumin (BSA) in PBS. After washing in PBS, tissue sections were incubated with biotinylated secondary antibodies (UltraVision Large Volume Detection System Anti-Polyvalent, HRP; LabVision) for 10 min. at room temperature. Subsequently, the slides were washed in PBS and incubated with streptavidin peroxidase (UltraVision Large Volume Detection System Anti-Polyvalent, HRP; LabVision) for 10 min. at room temperature. Immunoreactivity was developed using 3-amino-9-ethylcarbazole (AEC kit, catalogue no. TA-125-SA; LabVision) as chromogen. Sections were finally counterstained with haematoxylin, washed and mounted in an aqueous medium (VectaMount™ AQ; Vector Laboratories, Burlingame, CA, USA) and observed under a Leica DM4000 B microscope equipped with fully automated transmitted light axes (Leica Microsystems). Sections not exposed to primary antibodies were included as negative controls for antibody specificity.

### Immunofluorescence

Paraffin-embedded tissue sections (5 μm thick) were deparaffinized and boiled for 10 min. in sodium citrate buffer (10 mM, pH 6.0). Sections were washed in PBS, incubated in 2 mg/ml glycine for 10 min. to quench autofluorescence caused by free aldehydes, and then blocked for 1 hr at room temperature with 1% BSA in PBS. Slides were incubated overnight at 4°C with the following primary anti-human antibodies diluted in PBS with 1% BSA: mouse monoclonal anti-CD34 (1:50 dilution; Dako), rabbit polyclonal anti-c-kit/CD117 (1:300 dilution; catalogue no. A4502; Dako), rabbit polyclonal anti-CD31/platelet-endothelial cell adhesion molecule-1 (PECAM-1) (1:50 dilution; catalogue no. ab28364; Abcam), rabbit polyclonal anti-α-SMA (1:100 dilution; Abcam) and goat polyclonal anti-PDGFRα (1:100 dilution; catalogue no. AF-307-NA; R&D Systems, Minneapolis, MN, USA). For the double immunofluorescent staining, the sections were incubated with a mixture of monoclonal and polyclonal primary antibodies. The day after, the slides were washed three times in PBS and incubated for 45 min. at room temperature in the dark with a mixture of appropriate fluorochrome-conjugated secondary antibodies (Alexa Fluor 488-conjugated, Alexa Fluor 568-conjugated, or Rhodamine Red-X-conjugated IgG; Invitrogen, San Diego, CA, USA) diluted 1:200 in PBS with 1% BSA. Irrelevant isotype- and concentration-matched mouse, rabbit and goat IgG (Sigma-Aldrich, St. Louis, MO, USA) were used as negative controls. Cross-reactivity of secondary antibodies was tested in control experiments in which primary antibodies were omitted. In some sections, the nuclei were counterstained with 4′,6-diamidino-2-phenylindole (DAPI; Chemicon International, Temecula, CA, USA). Tissue sections were then mounted with an antifade aqueous mounting medium (Biomeda Gel Mount; Electron Microscopy Sciences, Foster City, CA, USA) and examined with a Leica DM4000 B microscope equipped with fully automated fluorescence axes (Leica Microsystems). Fluorescence images were captured with a Leica DFC310 FX 1.4-megapixel digital colour camera equipped with the Leica software application suite LAS V3.8 (Leica Microsystems).

## Results

The presence of TC was investigated in full-thickness biopsies of terminal ileum obtained from CD patients and controls. The morphological characteristics of the disease were assessed on haematoxylin–eosin- and Masson’s trichrome-stained slides. Sections obtained from disease-affected specimens showed the typical histopathological lesions of CD, whereas those from disease-unaffected specimens had a normal morphology.

Telocytes were identified by CD34 immunostaining and were easily distinguishable by other CD34-positive cells, such as CD31-positive vascular endothelial cells, and by c-kit-positive ICC. As we have previously reported 8, TC displayed different shapes and distribution according to their location in the different ileum wall layers.

**Figure 1:**
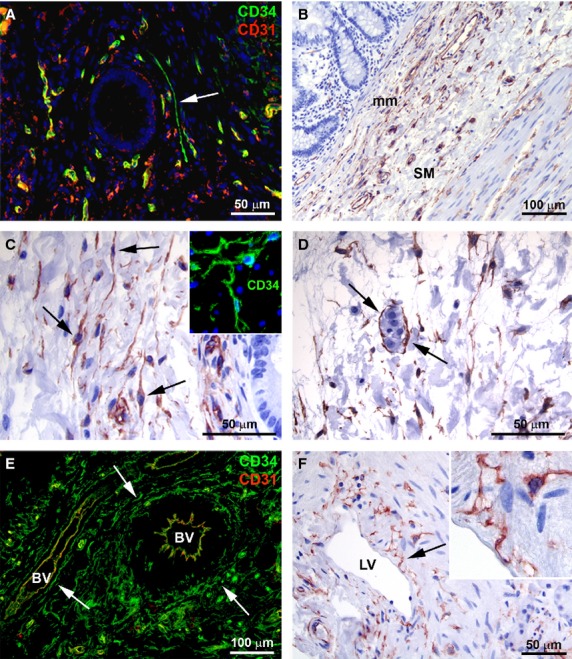
(A–F) Control ileal specimens. (A and E) Double immunofluorescence labelling for CD34 (green) and CD31 (red). In A, nuclei are counterstained with DAPI. (B–D and F) CD34 immunoperoxidase labelling with haematoxylin counterstain. (A) Mucosa. Note a telocyte (TC) near a glandular crypt (*arrow*). (B) TC are numerous in the muscularis mucosae and submucosa. (C) Muscularis mucosae. TC are mostly oriented parallel to each other and show a slender-nucleated body and two long processes (*arrows*). *Inset*: CD34 immunofluorescence labelling with DAPI counterstain. At higher magnification view, TC varicose processes (telopodes) are identifiable. (D) Submucosa. TC have a roundish body with two or three processes. Note a ganglia of the submucosal plexus surrounded by TC (*arrows*). (E) Submucosa. TC are particularly concentrated around large blood vessels, where they closely encircle the adventitial layer (*arrows*), and some TC are located among vascular smooth muscle cells within the vessel wall. Endothelial cells are CD34/CD31 double-positive, while TC are CD34-positive and CD31-negative. (F) Submucosa. TC are located around lymphatic vessels (*arrow*). *Inset*: Note the TC processes surrounding the discontinous basal lamina of a lymphatic vessel. mm: muscularis mucosae; SM: submucosa; BV: blood vessel; LV: lymphatic vessel. Scale bars are indicated in each panel.

In control samples, at the mucosa level, some TC surrounding the glandular crypts were observed in the lamina propria (Fig. 1A). In the muscularis mucosae, TC were numerous and mostly oriented parallel to each other (Fig. 1B and C). These cells showed a slender nucleated body and two long, thin and varicose processes, the telopodes, with alternating podomers and podoms (Fig. 1C and inset).

**Figure 2:**
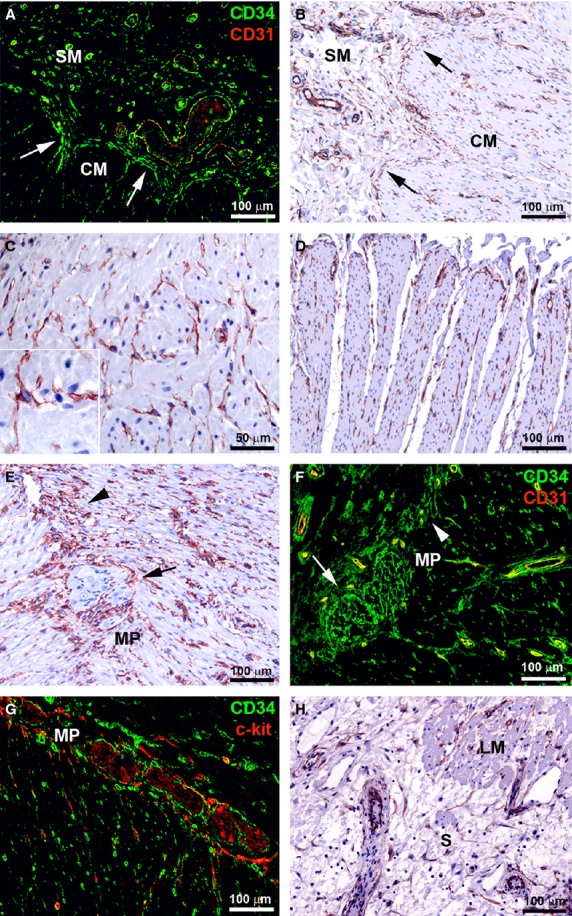
(A–H) Control ileal specimens. (A and F) Double immunofluorescence labelling for CD34 (green) and CD31 (red). (G) Double immunofluorescence labelling for CD34 (green) and c-kit (red). (B–E and H) CD34 immunoperoxidase labelling with haematoxylin counterstain. (A and B) Submucosa and circular muscle layer. Telocytes (TC) form a continuous layer bordering the circular muscle layer (*arrows*). (C) Muscularis propria. TC form a network around smooth muscle fibres. *Inset*: At higher magnification view, intramuscular TC display a roundish or oval body and three or four varicose processes. (D) Muscularis propria. TC are embedded in the connective tissue between muscle bundles. (E and F) Myenteric plexus. TC form a network enveloping the ganglia (*arrows*) and the enteric strands in intergangliar region (*arrowheads*). TC processes are intermingled with ganglion cells. (G) Myenteric plexus. TC and interstitial cells of Cajal (ICC) encircle together the myenteric plexus. (H) Subserosa. TC are visible around vessels, nerves and among adipocytes. SM: submucosa; CM: circular muscle layer; MP: myenteric plexus; S: subserosa; LM: longitudinal muscle layer. Scale bars are indicated in each panel.

At the submucosa level, TC were observed among the collagen and elastic fibres and around the ganglia of the submucosal plexus (Fig. 1D). Moreover, TC were particularly concentrated around large blood vessels, where they closely encircled the adventitial layer (Fig. 1E). Some TC were also located among vascular smooth muscle cells within the vessel wall (Fig. 1E). In agreement with previous reports 8, TC were CD34-positive and CD31-negative, and therefore they could be clearly distinguished from CD31/CD34 double-positive endothelial cells (Fig. 1E). A network of TC was also present around lymphatic vessels (Fig. 1F). In the submucosa, TC showed a roundish body with two or three processes (Fig. 1D) and formed an almost continuous layer bordering the circular muscle layer (Fig. 2A and B).

In the muscularis propria, TC were distributed around and within the circular and longitudinal muscle layers and in the myenteric plexus region (Fig. 2A–G). They displayed a roundish or oval body and three or four processes running in every direction around the smooth muscle fibres, likely contacting each other (Fig. 2C and inset). Moreover, some TC were embedded in the connective tissue between muscle bundles, running parallel to them (Fig. 2D). Telocytes formed a network enveloping the ganglia of the myenteric plexus, and their processes extended intermingling with neuronal and glial cells (Fig. 2E and F). They were also present along the enteric strands of the intergangliar region (Fig. 2E and F). CD34/c-kit double immunolabelling demonstrated that CD34-positive TC and c-kit-positive ICC formed two distinct networks that together encircled the myenteric plexus (Fig. 2G).

In the subserosa, TC ran in the connective strands and were located around nerves and vessels and among adipocytes (Fig. 2H).

In disease-unaffected ileal specimens from CD patients, the distribution of TC was similar to that observed in control samples (data not shown). As expected, disease-affected specimens appeared severely and patchy damaged. Indeed, CD does not spread in a continuous manner, but it is characterized by ‘skip lesions’, with microscopically affected areas alternating with spared ones. Similarly, in the affected CD specimens we found that TC were rare or mostly disappeared in the areas displaying histopathological damages, while their distribution was similar to controls in the healthy-looking areas.

**Figure 3:**
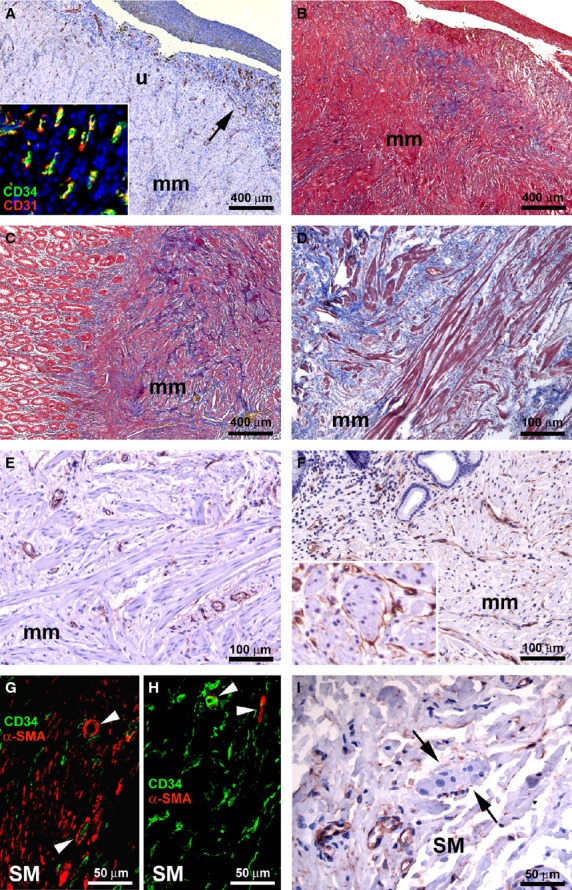
(A–G and I) Affected ileal specimens from Crohn’s disease (CD) patients. (H) Control ileal specimens. (A, E, F and I) CD34 immunoperoxidase labelling with haematoxylin counterstain. (B–D) Masson’s trichrome staining. (G and H) Double immunofluorescence labelling for CD34 (green) and α-smooth muscle actin (α-SMA) (red). (A and B) Mucosa and submucosa. Severely damaged tissue with a haemorrhagic ulcer. (A) Telocytes (TC) are not visible, while many CD34-positive microvessels are present (*arrow*). *Inset*: CD34/CD31 double immunofluorescence labelling with DAPI counterstain. At higher magnification view, many CD34/CD31-positive microvessels are evident. (B) The inflammation extends to the muscularis mucosae and submucosa, with a pattern of incoming fibrosis. (C) Mucosa, muscularis mucosae and submucosa. The thickened and fibrotic muscularis mucosae extends to the circular muscle layer, replacing the normal submucosa. (D) Muscularis mucosae. Muscle cells are disarranged and separated by fibrosis. (E and F) Muscularis mucosae. Most of the TC disappear (E) and the few remaining ones are located among smooth muscle cells (F). *Inset*: Higher magnification view displaying TC around smooth muscle bundles. (G and H) Submucosa. (G) In affected CD specimens, CD34/α-SMA double immunolabelling highlights the loss of TC and the presence of many α-SMA-positive spindle-shaped myofibroblasts. (H) In control specimens, many CD34-positive TC are present, while myofibroblasts are not detectable. In both CD and control specimens, vascular pericytes display α-SMA positivity (*arrowheads*). (I) Submucosa. Few or no TC are present around submucosal ganglia (*arrows*). u: ulcer; mm: muscularis mucosae; SM: submucosa. Scale bars are indicated in each panel.

In severely affected specimens, we observed ulcers, often with haemorrhagic rim and frequently adjacent to a normal or minimally inflamed mucosa. Here, TC were not visible, whereas several newly formed CD34-positive microvessels were frequently observed under the haemorrhagic ulcers (Fig. 3A). This was further confirmed by double immunolabelling evidencing CD34/CD31-positive endothelial cells (Fig. 3A inset). In the spared areas of the mucosa, where glandular crypts appeared healthy, some TC were present as observed in control specimens.

**Figure 4:**
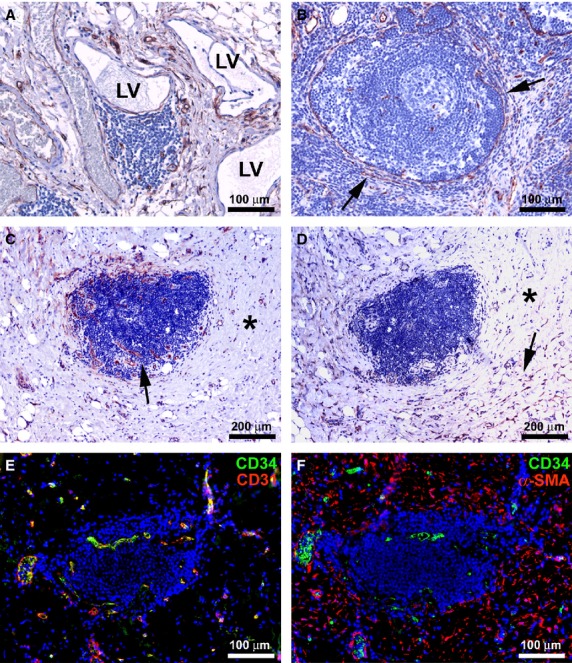
(A–F) Affected ileal specimens from Crohn’s disease (CD) patients. (A–D) Immunoperoxidase labelling for CD34 (A–C) and α-smooth muscle actin (α-SMA) (D) with haematoxylin counterstain. (E and F) Double immunofluorescence labelling for CD34 (green) and CD31 or α-SMA (red) with DAPI counterstain. (A) Submucosa. Telocytes (TC) encircle blood vessels and enlarged lymphatic vessels. (B) A reactive lymphoid aggregate appears entirely encircled by TC (*arrows*). (C and D) A lymphoid aggregate, completely embedded in the fibrotic extracellular matrix (C, *asterisk*), is surrounded by few TC and display many newly formed microvessels (C, *arrow*). The presence of fibrosis (D, *asterisk*) is confirmed by numerous α-SMA-positive myofibroblasts (D, *arrow*). (E and F) CD34/CD31 and CD34/α-SMA double immunolabelling confirms the absence of TC and the presence of numerous microvessels and myofibroblasts. LV: lymphatic vessel. Scale bars are indicated in each panel.

The inflammation was often diffuse, extending to the muscularis mucosae and submucosa, with a pattern of incoming fibrosis as revealed by Masson’s trichrome staining (Fig. 3B). The muscularis mucosae appeared duplicated or markedly thickened and fibrotic, particularly at sites of present or previous mucosal ulceration. The thickened muscularis mucosae could even extend to the circular muscle layer, replacing the entire submucosa. Smooth muscle cells were disarranged and separated by the fibrotic extracellular matrix, as observed with Masson’s trichrome staining (Fig. 3C and D). In the altered muscularis mucosae, most of the TC disappeared and the few remaining ones were located among smooth muscle cells (Fig. 3E and F and inset). In the fibrotic submucosa, CD34/α-SMA double immunolabelling not only demonstrated the loss of TC, but it also revealed the presence of many α-SMA-positive spindle-shaped myofibroblasts, which are believed to be mostly responsible for the excessive production of collagen fibres during the fibrotic process (Fig. 3G). On the contrary, in the submucosa of control specimens many CD34-positive TC were present, while myofibroblasts were not detected (Fig. 3H). Indeed, in control sections α-SMA positivity was observed only in vascular pericytes (Fig. 3H). In affected CD specimens, few TC were observed around the remaining submucosal ganglia (Fig. 3I). Moreover, TC were still present around blood and enlarged lymphatic vessels (Fig. 4A). In both muscularis mucosae and submucosa, the remaining TC exhibited morphological features similar to those observed in control and unaffected CD samples. Despite the architectural derangement of the muscularis mucosae and submucosa, TC were preserved at the submucosal border of the circular muscle layer. Some reactive lymphoid aggregates appeared entirely encircled by TC (Fig. 4B). Conversely, the lymphoid aggregates that appeared completely embedded in the fibrotic extracellular matrix were surrounded by few TC and displayed many newly formed microvessels (Fig. 4C). Myofibroblasts were clearly visible in the fibrotic tissue around such lymphoid aggregates (Fig. 4D). CD34/CD31 and CD34/α-SMA double immunolabelling confirmed both the absence of TC and the presence of numerous microvessels and myofibroblasts (Fig. 4E and F).

**Figure 5:**
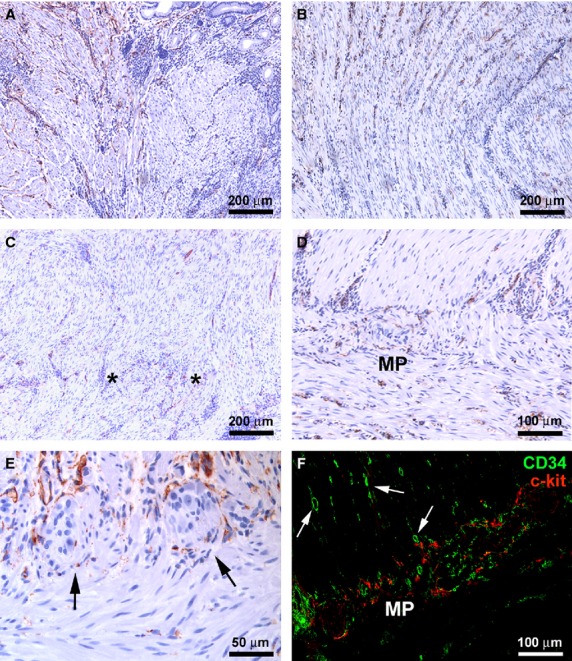
(A–F) Affected ileal specimens from Crohn’s disease (CD) patients. (A–E) CD34 immunoperoxidase labelling with haematoxylin counterstain. (F) Double immunofluorescence labelling for CD34 (green) and c-kit (red). (A and B) Muscularis propria. Telocytes (TC) maintain their morphology and distribution around and along smooth muscle fibres in a spared area of muscularis propria (A, left side; B, upper side), while they are not present in the close damaged area (A, right side; B, lower side). (C–F) Myenteric plexus. (C) Irregular distribution of ganglia (*asterisks*) between the circular and longitudinal muscle layers. (D and E) The TC network around ganglia (*arrows*) and enteric strands in intergangliar regions is discontinuous or even completely absent. (F) A discontinuous network of CD34-positive TC and c-kit-positive ICC is present around myenteric plexus ganglia. TC and ICC are almost completely absent in muscle layers, while many CD34-positive microvessels (*arrows*) are evident. MP: myenteric plexus. Scale bars are indicated in each panel.

In the muscularis propria, we found areas with a preserved architecture of the circular and longitudinal muscle layers close to areas displaying severe histopathological damages. In the spared areas TC maintained their morphology and distribution around and along muscle bundles and fibres, while they disappeared in the damaged ones (Fig. 5A and B). A patchy involvement of the myenteric plexus was also observed. Telocyte distribution around myenteric ganglia and enteric strands was similar to control specimens in the healthy-looking areas. In the most severely damaged muscularis propria, a disorganization of the myenteric plexus characterized by an irregular distribution of ganglia between the circular and longitudinal muscle layers was observed (Fig. 5C and D). Here, the TC network around ganglia and enteric strands of intergangliar regions was discontinuous or even completely absent (Fig. 5C–F). Moreover, CD34/c-kit double immunolabelling revealed that the loss of TC was paralleled by the loss of the interstitial cells of Cajal network (Fig. 5F).

**Figure 6:**
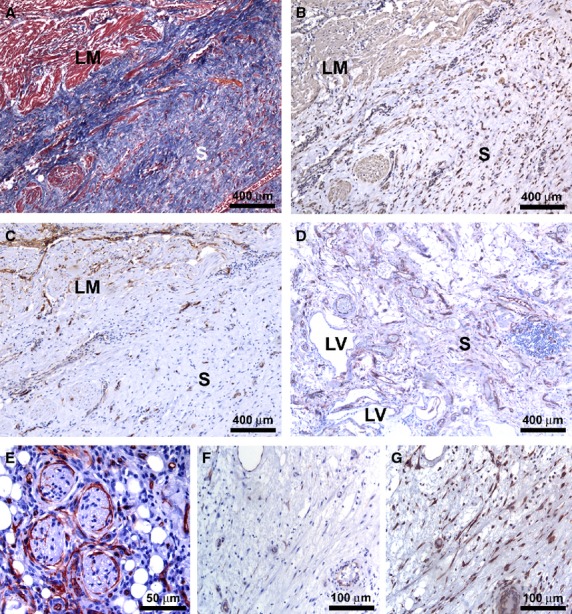
(A–G) Affected ileal specimens from Crohn’s disease (CD) patients. (A) Masson’s trichrome staining. (B–G) Immunoperoxidase labelling for α-smooth muscle actin (α-SMA) (B and G) and CD34 (C–F) with haematoxylin counterstain. (A) At the border with the subserosa, longitudinal muscle bundles are separated by inflammatory infiltrate and fibrous connective tissue. In consecutive immunostained sections, many α-SMA-positive myofibroblasts are visible (B) while CD34-positive telocytes (TC) are absent (C). (D and E) In areas of the subserosa displaying inflammatory infiltration and incoming fibrosis, TC are not visible around vessels (D), but they are still present around nerves (E). (F and G) In areas of the subserosa with settled fibrosis, TC are rare or completely absent (F), while numerous α-SMA-positive myofibroblasts are observed (G). LM: longitudinal muscle layer; LV: lymphatic vessel; S: subserosa. Scale bars are indicated in each panel.

At the border with the subserosa, longitudinal muscle bundles were separated by inflammatory infiltrate and fibrous connective tissue (Fig. 6A). Several α-SMA-positive myofibroblasts were visible, whereas TC could not be observed (Fig. 6B and C). In the areas of the subserosa displaying inflammatory infiltration and incoming fibrosis, TC were not found around vessels, while they were still present in the connective strands and around nerves (Fig. 6D and E). On the contrary, in the areas of settled fibrosis, TC were rare or completely absent, whereas numerous α-SMA-positive myofibroblasts were observed (Fig. 6F and G).

**Figure 7:**
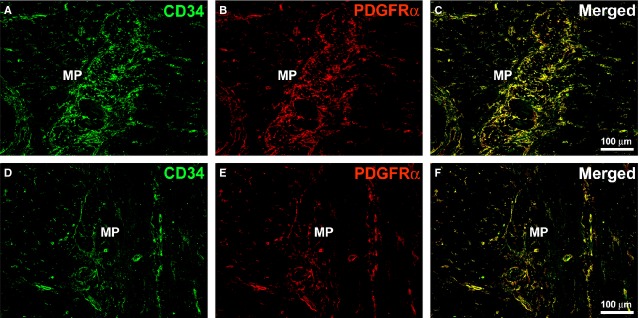
(A–C) Control ileal specimens. (D–F) Affected ileal specimens from Crohn’s disease (CD) patients. (A–F) Double immunofluorescence labelling for CD34 (green) and platelet-derived growth factor receptor α (PDGFRα) (red). In the ileal wall, all CD34-positive telocytes (TC) are also PDGFRα-positive. (A–C) In control specimens, TC form a broad network surrounding the myenteric plexus. (D–F) In severely damaged areas of affected CD specimens, the TC network around the myenteric plexus is discontinuous or even absent. MP: myenteric plexus. Scale bars are indicated.

As it has been recently demonstrated that TC express PDGFRα in the human GI tract 8, we also performed double immunostaining for CD34 and PDGFRα. Consistent with previous findings 8, all CD34-positive TC were also PDGFRα-positive in our ileal wall sections (Fig. 7). This analysis confirmed the loss of TC in disease-affected specimens from CD patients. Indeed, CD34/PDGFRα double-positive TC were severely reduced in the damaged areas of affected CD samples compared with controls (Fig. 7).

## Discussion

In this study, we investigated for the first time the presence and distribution of TC in the ileal wall of patients with CD who underwent surgery for intestinal obstructive stenosis.

Conventionally, cells described in the stroma of the intestinal wall are fibroblasts, mast cells, plasma cells, eosinophils, macrophages and ICC, the latter being considered as the pacemaker cells that regulate GI rhythmicity. Recently, the presence of a new interstitial cell type, named TC, has been reported in a variety of cavitary and non-cavitary organs (http://www.telocytes.com) 13–36. To date, a number of studies have described TC in the GI tract of humans and laboratory mammals 7–17. TC are characterized by very long prolongations, called telopodes, which form a three-dimensional (3-D) network close to immune cells, smooth muscle cells, blood and lymphatic vessels and nerves 18. By immunohistochemistry, CD34 labelling is the first and the best available choice to identify TC 7,18.

We herein examined disease-affected and -unaffected specimens of resected ileal segments from CD patients in order to evaluate the presence and distribution of TC in comparison with specimens obtained from controls. In particular, we identified TC by CD34 and PDGFRα immunolabelling, and we also used CD34/CD31, CD34/c-kit and CD34/α-SMA double immunolabelling to clearly distinguish TC from endothelial cells, ICC and myofibroblasts, respectively. In control specimens, TC were observed in all the ileal wall layers, from the mucosa to the subserosa, and they showed a slender-nucleated body and two or more telopodes, confirming the literature description 7–18. According to the different wall layers, they run parallel to each other and/or formed a network, as we observed in the muscularis propria and at the myenteric plexus. The role of these cells in the gut is still not fully understood, but some relevant and potential functions have been proposed. In particular, taking into account the 3-D network of telopodes and their strategic position in between immune cells, smooth muscle cells, blood and lymphatic vessels and nerve endings, it has been suggested that TC may be involved in intercellular signalling, immune surveillance and neurotransmission 8–18.

In CD, the most peculiar histopathological features of the affected intestinal wall segments are represented by discontinuous signs of inflammation and fibrosis, also indicated as ‘skip lesions’. In disease-unaffected specimens from CD patients that appeared morphologically comparable to control samples, TC exhibited a distribution similar to controls throughout the different ileal wall layers. Conversely, in sections from disease-affected specimens TC disappeared, particularly in areas where severe fibrosis and architectural derangement of the intestinal wall were observed.

In severe cases, the muscularis mucosae appeared markedly thickened because of a prominent fibrosis involving also the underlying submucosa. Here, the few remaining TC were located among the smooth muscle bundles and cells. We suppose that the fibrotic process may entrap TC in a poorly permeable extracellular matrix, with consequent cell sufferance, and may also alter their spatial relationships with the other connective cells, immune and muscle cells possibly impairing their interconnecting role. On the other hand, the progressive loss of TC in the CD intestinal wall could also contribute to the altered 3-D organization of the extracellular matrix and to the progression of fibrosis, as it has been recently proposed in the skin of systemic sclerosis patients 37. Interestingly, in a recent study TC were found to be decreased during experimental myocardial infarction, particularly in fibrotic areas, and transplantation of cardiac TC in the infarcted and border zones of the heart decreased the infarction size and improved myocardial function 38. Taken together, these data clearly point to a broad involvement of TC in the fibrotic remodelling of multiple organs in different diseases.

In affected CD samples, some reactive lymphoid aggregates, especially those surrounded by a prominent and diffuse inflammatory infiltrate, were completely encircled by TC, likely in the attempt to limit their spreading in the connective tissue. Similar patterns were described in human heart amyloidosis, where amyloid deposits were located in interstitial recesses surrounded by long and slender TC processes 39. Instead, TC disappeared when the lymphoid aggregates were entrapped by fibrotic tissue containing many myofibroblasts.

In the muscularis propria, we found a severe derangement of muscle bundles with loss of the normal organization in the circular and longitudinal layers. It is noteworthy that the TC network could be still observed among smooth muscle fibres in some areas close to others where TC were completely absent. This severe architectural derangement involved also the myenteric plexus, where a discontinuous network of TC was observed around ganglia and enteric strands. In addition, CD34/c-kit double labelling revealed that TC disappearance was paralleled by the loss of the ICC network. The motility of the GI tract involves complex processes that require the structural integrity and functionality of each cellular element involved. Mechanical activity of the smooth muscle cells is in part controlled by the enteric nervous system and ICC. In the gut, the TC 3-D network might play a mechanical and supporting role being resistant to and deformable following intestine movements 7–8. Moreover, as some of the intramuscular TC and ICC seem to be part of a unique network, in which ICC only/preferentially are in close contact with nerve endings, it has been suggested that TC might play a role in neurotransmission in the gut, possibly contributing to spread the slow waves generated by the ICC 8–18. Evidence of dysmotility at different levels of the GI tract has been reported in CD 6, and this has been related to a severe damage of the enteric neural structures and a reduction in the ICC population 6–25. On the basis of our findings, we can hypothesize that the loss of the TC network in the muscularis propria and myenteric plexus might contribute to intestinal dysmotility in CD patients.

Even at the subserosa level, we found that TC were still present in areas with prominent inflammatory process, whereas they disappeared in areas with settled fibrosis, reflecting the ‘skip lesions’ pattern observed in the other ileal wall layers.

In conclusion, our data clearly demonstrate that TC are involved in the pathological processes of CD. Several roles have been suggested for the TC, most of which are believable and not mutually exclusive, though none of them has been certainly proved yet. Telocytes may have a role (*i*) as mechanical support in the connective tissue 18; (*ii*) in intercellular signalling between stromal cells, immune cells, smooth muscle cells, capillary vessels and nerve bundles, both through cell-to-cell contacts and release of paracrine mediators 15–40; (*iii*) in the neurotransmission, even considering their relationship with the ICC 8–18. In line with these proposed functions, our results suggest that in CD the loss of TC might be implicated in the derangement of the intestinal wall architecture, gut dysmotility and impaired immune surveillance. In the unaffected ileal segments and in the spared areas of the affected CD specimens, TC preserved their morphology and distribution likely maintaining their supposed roles. Further ultrastructural and functional studies are necessary to better clarify the role of TC loss in CD pathophysiology.
